# Menu Dilemmas: An Integrated Assessment of the Nutritional Quality, Environmental Impact, and Cost of Vegan, Vegetarian, and Meat-Based Versions of Meals

**DOI:** 10.3390/nu17091569

**Published:** 2025-05-02

**Authors:** Berill Takacs, Anastasia Z. Kalea, Aiduan Borrion

**Affiliations:** 1 UCL Department of Civil, Environmental and Geomatic Engineering, University College London, London WC1E 6BT, UK; a.borrion@ucl.ac.uk; 2 Centre for Urban Sustainability and Resilience, University College London, London WC1E 6BT, UK; 3 UCL Division of Medicine, University College London, London WC1E 6BT, UK; a.kalea@ucl.ac.uk; 4 Institute of Cardiovascular Science, University College London, London WC1E 6DD, UK

**Keywords:** sustainability, meals, plant based, vegan, life cycle assessment, nutrient profiling, nutrient rich food index, environmental nutrition

## Abstract

**Background/Objectives**: Adopting sustainable dietary patterns is essential for addressing environmental sustainability and improving public health outcomes. However, food service providers and consumers often face challenges in making informed choices due to a lack of information on the environmental, nutritional, and cost implications of different meal options. The aim of this paper was to provide an integrated assessment of the nutritional quality, environmental impact and cost of vegan, vegetarian, and meat-based versions of four popular meals (lasagne, chilli, teriyaki, and curry) offered in the lunch service of a university food service establishment in London, UK. **Methods**: In this study, real recipes from the food service provider were analysed. The nutritional quality of meals was evaluated using the Nutrient Rich Food Index (NRF 9.3 and 17.3), the environmental impact was assessed using life cycle assessment (LCA), and the cost was calculated using recipe costing. Results were normalised using the min–max method, and recipes were ranked relative to each other based on their final nutritional quality, environmental impact and cost scores using a normalised integrated scoring method to identify which recipe version of meals was the most optimal when considering environmental sustainability, nutrition, and cost simultaneously. **Results**: The integrated assessment revealed that vegan recipe versions of meals made with whole foods consistently outperformed their meat-based counterparts across all three criteria—environmental impact, nutritional quality, and cost—ranking highest in environmental sustainability and nutrition while also being more cost-effective, regardless of cuisine or dish type. **Conclusions**: These findings suggest that shifting towards plant-based recipes made with whole-foods (e.g., vegetables, legumes, etc.) can improve micronutrient intake, reduce environmental impact, and lower costs, thus supporting sustainable dietary transitions and public health.

## 1. Introduction

Ensuring sustainable and healthy food production and consumption remains a significant challenge for the global food system. Our current food system is a major driver of climate change [1], environmental degradation and biodiversity loss [2,3], with meat, eggs, and dairy being the main drivers of impacts [3,4]. Meanwhile, current dietary patterns are also causally linked with malnutrition, obesity and non-communicable diseases such as cardiovascular disease, diabetes and cancer, which account for 75% of all deaths globally [5]. Micronutrient deficiencies remain a widespread issue and can impair immune function and increase susceptibility to infectious and chronic diseases [6,7]. Consequently, it is important to help populations achieve adequate nutrient intakes without compromising planetary health [8].

Food-based approaches that encourage greater consumption of fruits, vegetables, whole grains, and pulses have been identified as viable and cost-effective ways to both prevent micronutrient deficiencies and promote healthy and sustainable diets [8,9]. Several studies have already confirmed the environmental benefits of plant-based diets and meals [10,11,12,13,14,15] and compared the nutritional adequacy of different types of dietary patterns, such as omnivore, vegetarian and vegan diets [16,17,18].

Plant-based diets not only tend to be better for the environment but also for health [19,20,21] and are associated with lower inflammation, reduced risks of chronic diseases, and decreased all-cause mortality [22,23,24,25]. However, as with any diet, appropriate planning is needed to avoid the risk of deficiencies in certain nutrients such as calcium, iron, zinc, and vitamin B12 [26]. Importantly, nutrient inadequacies are not exclusive to plant-based diets and can be found across all dietary patterns, highlighting the need to improve diet quality by including more diverse, nutrient-dense plant-based foods in diets [20]. In the United Kingdom (UK), only 33% of adults meet the ‘5 A Day’ recommendation for fruit and vegetable consumption. Adults have an average fibre intake of 19.7 g per day, falling short of the recommended 30 g per day [27]. Young adults in the UK also have significantly lower intakes of several micronutrients, including vitamin A, riboflavin, folic acid, calcium, magnesium, potassium, iodine, and copper, than is recommended [28].

Although there has been increasing focus on creating tools and methods to promote sustainable food consumption [29,30,31], there is still a need for harmonised and integrated assessments that simultaneously evaluate various impacts (e.g., environmental, nutritional, and economic) of food choices [32,33]. While diet-level assessments provide important insights into long-term dietary patterns and nutrient adequacy, meal-level analyses allow for targeted interventions to address specific nutrient deficiencies and offer more precise and immediate strategies for sustainable and healthy dietary adjustment [34].

The aim of this study was to provide an integrated assessment of the nutritional quality, environmental impact and cost of vegan, vegetarian, and meat-based recipe versions of four popular meals: lasagne, chilli, teriyaki, and curry. Using a real-life case study from a university food service establishment in London, UK, this research assessed actual recipes. The selected four meals were the top-selling items on the lunch menu. Given the popularity of these meals, there was a strong interest in optimising these meals for environmental impact, nutritional quality, and cost. By comparing different recipe variations of these meals using nutrient profiling (Nutrient Rich Food Index), Life Cycle Assessment (LCA), and recipe costing, this study explores the impact of ingredient substitutions at the meal level in a real-world food service setting. Poorly planned meals and menus of any type—vegan, vegetarian, or omnivorous—can lead to nutritional imbalances; therefore, it is important to examine how specific ingredient changes in meals may impact the nutritional quality as well as environmental sustainability and cost of individual meals.

## 2. Materials and Methods

### 2.1. Study Overview

In this study, an integrated assessment of the environmental impact, nutritional quality, and cost of vegan, vegetarian, and meat-based versions of four popular meals (lasagne, chilli, teriyaki, and curry) served by a university food service provider in London, UK, was conducted. Lasagne, chilli and curry meals were not only among the most popular meals on offer but are also some of the most well-known dishes consumed in the UK and elsewhere [35], making the findings relevant to a wide range of audiences.

**Figure 1:**
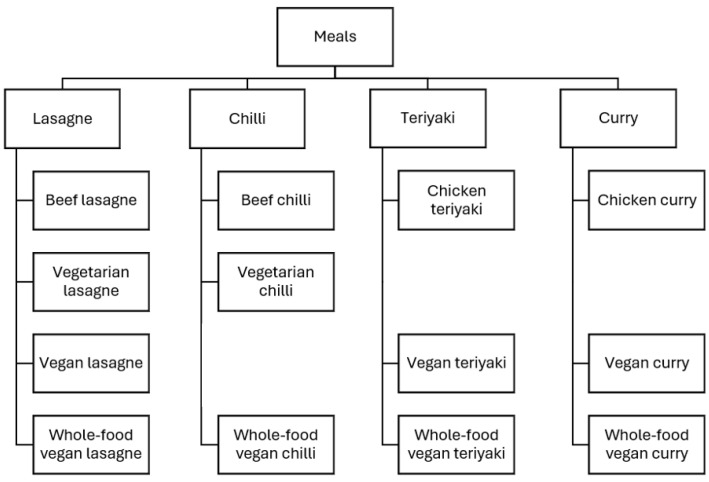
Flowchart illustrating the categorisation of meals and their recipe variations analysed in this study.

In total, 13 recipes were analysed (see Figure 1), of which six were animal-based (n = 4 meat-based; n = 2 vegetarian) and seven were plant-based (n = 3 vegan; n = 4 whole-food vegan). The meat-based recipes served as the baseline recipe, and the impact of ingredient substitutions and recipe modifications was assessed across three criteria: nutritional quality, environmental impact, and recipe cost. The recipes analysed in this study were real recipes provided by the university food service establishment and were used as a case study to examine how replacing meat, dairy and animal-based ingredients in popular dishes affects the environmental impact, nutritional quality and cost of meals. In the vegetarian recipes, meat was replaced with a vegetarian meat substitute (e.g., Quorn). In vegan recipes, meat and dairy were replaced either with vegan substitutes (e.g., vegan mince, vegan cheese, etc.) or with whole or minimally processed ingredients such as legumes, vegetables and/or tempeh in the whole-food vegan recipes. A distinction was made between ‘vegan’ and ‘whole-food vegan’ recipes where possible, given the potential disparity in their nutritional quality [34,36,37,38]. All meals and their recipe variations analysed in this study are shown in Appendix A (Table A1) with their ingredients, serving weights and energy content per meal. Ingredient substitutions, such as vegan cheese, were determined by the food service provider, who supplied the recipe cards and procurement data. Consequently, the analysis was based on the options and brands specified in their recipe cards.

First, the nutritional quality, environmental impact and costs of each recipe were assessed individually (see Section 2.2, Section 2.3 and Section 2.4) and calculated per portion of served meal, which varied by recipe. The results were then integrated (see Section 2.5) to identify which recipe variations were the most sustainable based on the three criteria assessed in this study.

### 2.2. Nutritional Quality Assessment

The nutritional composition of meals was analysed using Nutritics (v5.63), which used the McCance and Widdowson’s Composition of Foods Integrated Dataset (CoFID) as a primary data source for the UK. Once the nutritional composition of each meal was obtained, a well-established nutrient profiling model, the Nutrient Rich Food (NRF) index, [39,40,41] was employed to evaluate and compare the nutrient density of each recipe. In this study, the NRF 9.3 index [39] was used, which is based on nine nutrients to encourage (protein, fibre, vitamin A, vitamin E, vitamin C, calcium, magnesium, iron, and potassium) and three nutrients to limit (saturated fat, added sugar, and sodium).

Since the NRF index was originally developed to assess nutrients of concern of American adults, it does not include several of the nutrients of public health concern for UK adults (e.g., riboflavin, folic acid, iodine, and copper) [28]. To address this gap, a new version of the NRF index, the NRF 17.3, was developed in which those nutrients identified as of concern for UK adults were also included. In addition, important micronutrients such as zinc and selenium were also included in the NRF 17.3, as these play critical roles in mediating inflammatory response and help maintain and improve immune function, which is especially important in the wake of pandemics [42]. Thus, the NRF 17.3 index included 17 nutrients to encourage: protein, fibre, seven vitamins (A, B1, B2, B9, B12, C, and E) and eight minerals (K, Ca, Mg, Fe, I, Cu, Zn, and Se). The nutrients to limit remained unchanged and were saturated fat, added sugar, and sodium. The benefit of using these two versions of the NRF index is that the use of the original NRF 9.3 index allows for the comparison of results with other studies, while the modified NRF 17.3 allows for a better understanding of how well recipes perform in meeting the recommended nutrients of concern for UK adults, which was the target population in this study.

Nutrient density was calculated relative to one portion of cooked meal, as indicated by the recipe cards. The NRF n.3 scores were calculated for each recipe as the sum of values of the n qualifying nutrients to encourage minus the sum of values of the three nutrients to limit. Reference Nutrient Intakes (RNI) for the nutrients were based on the guidelines set by the UK government [43,44]. In the NRF models, values were capped at 100% for positive nutrients so that excessive intakes (i.e., over the RNI) of a single nutrient would not result in a disproportionately high NRF score [40]. Capping nutrients at 100% also prevents one nutrient from being compensated for with the inadequate intake of other nutrients.

### 2.3. Environmental Impact Assessment

The environmental impacts of meals were derived from the work of Takacs et al. [15], which used the standard life cycle assessment (LCA) methodology (ISO 14040 and ISO 14044 [45,46]) to estimate the environmental impacts of the meals and recipe variations analysed in this study. The environmental impacts were based on four impact categories: global warming potential (GWP), freshwater eutrophication potential (FEP), terrestrial acidification potential (TAP), and water depletion potential (WDP). The stages included in the system boundary were ingredient production, transportation of ingredients from site of production to site of processing and/or central kitchen, storage of ingredients in walk-in chillers and freezers used at the central kitchen, and meal preparation (cooking). Data for the LCA, including inputs for ingredient production and processing and data on energy and transport, were sourced from the literature and the Ecoinvent 3.6 database (2019). For a detailed explanation of the LCA methodology, please refer to the original study [15].

### 2.4. Recipe Costing

Recipe costing was used to compare the costs of different recipe variations by calculating the price of each ingredient based on its unit cost and the quantity used in each recipe. Data on ingredients and quantities used, brand, pack size and product code were collected from the recipe cards, which were provided by the food service provider to calculate recipe costs. Ingredient prices were sourced from the website of the wholesaler that was used by the food service provider (as of March 2025). For each ingredient, the cost was calculated based on the actual amount used in the recipe, rather than the full price of the unit purchased.

### 2.5. Integrated Assessment

To enable direct comparison between recipes across nutritional quality, environmental impact, and cost, the results of each individual assessment were normalised separately using the min–max technique. This method scaled results for each criterion between 0 and 1, with the worst option for each attribute receiving a score of 0 and the best option receiving a score of 1. The remaining options were assigned scores between 0 and 1, depending on how they compared to the worst and best options.

The environmental impact assessment included four impacts—climate impact, water pollution (eutrophication), acidification, and water depletion—which were weighted equally to generate a single normalised environmental impact score for each recipe. The results of the nutritional quality assessment, which was based on the nutrient density score (NRF 17.3) and overall nutritional composition, were normalised into one final nutritional quality score, with each of the 20 nutrients in the NRF 17.3 given equal weight. Recipe costs were likewise normalised to a single recipe cost score.

Following normalisation, recipes were ranked relative to each other based on their final environmental impact score, final nutritional quality score, and recipe cost score. The final sustainability ranking integrated these normalised scores, with equal weight assigned to each score, based on the assumption that the nutritional quality, environmental impact, and recipe costs are equally important in determining the overall sustainability of meals. However, to test whether different weightings assigned to each criterion would affect the final ranking, a weighted decision matrix with six different weighting scenarios was also created (see Appendix B).

## 3. Results

### 3.1. Nutritional Quality

#### 3.1.1. Nutrient Density of Recipes

**Figure 2:**
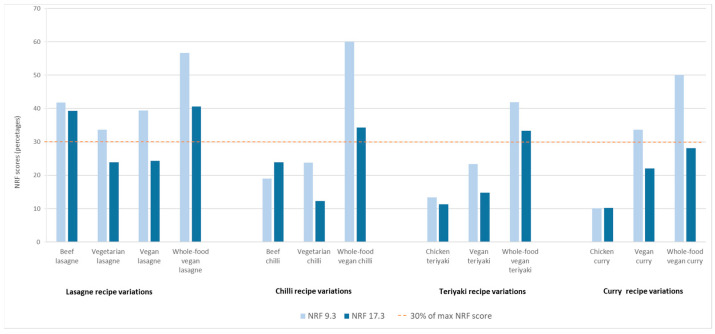
Comparison of the nutrient density of meat-based, vegetarian, vegan and whole-food vegan recipe variations of lasagne, chilli, teriyaki, and curry meals according to the NRF 9.3 and NRF 17.3 scores. Note: Since each NRF score has a different maximum score (600 for NRF 9.3 and 1400 for NRF 17.3), the NRF scores are expressed in percentages instead of actual scores. Actual NRF scores are available in Appendix C (Table A2).

The nutrient density of lasagne, chilli, teriyaki and curry meals and their meat-based, vegetarian, vegan and whole-food vegan recipe variations calculated according to the NRF 9.3 and 17.3 indices are shown in Figure 2. Although there were slight differences in the final nutrient density scores depending on the NRF model used, the overall trends were consistent across meals and their recipe variations.

Based on the assumption that a main meal should provide approximately 30% of the daily reference nutrient intakes [47], an ideal recipe should have an NRF score equal to approximately 30% of the maximum NRF score. In this study, eight out of thirteen recipes met this criterion and had NRF 9.3 scores above 30% of the maximum score (>180 out of 600). These included all four whole-food vegan recipes, all lasagne recipes, and the vegan curry. When using the NRF 17.3 model, only four recipes (beef lasagne, whole-food vegan lasagne, whole-food vegan chilli and whole-food vegan teriyaki) had NRF 17.3 scores that reached at least 30% of the maximum score (>420 out of 1400). These results suggest that when more nutrients are included in the NRF score, it may be more difficult, but not impossible, for recipes to meet the 30% threshold.

#### 3.1.2. Nutritional Composition of Recipes

##### Lasagne Recipes

According to the NRF 17.3 model, the most nutrient-dense lasagne recipes were the whole-food vegan lasagne (NRF 17.3 score 569), followed by the beef lasagne (NRF 17.3 score 550), vegetarian lasagne (NRF 17.3 score 335) and vegan lasagne (NRF 17.3 score 340). All lasagne recipes provided more than 30% of the daily reference nutrient intake (RNI) of protein. The beef lasagne provided 49 g of protein (98% of daily RNI), the vegetarian lasagne provided 25 g (50% of RNI), the vegan lasagne provided 23 g (46% of RNI) and the whole-food vegan lasagne provided 19 g of protein (38% of RNI). Only the whole-food vegan lasagne exceeded 30% of the recommended fibre intake (14 g, 47% of RNI), while the other lasagne recipes provided 30% (vegan), 27% (vegetarian), and 13% (beef) of RNI. For saturated fat, only the whole-food vegan was below 30% of RNI. In contrast, the beef lasagne provided 55%, the vegetarian 40% and the vegan 30% of RNI for saturated fat. The whole-food vegan lasagne also provided the highest amounts of thiamine (B1) (0.5 mg, 45% of RNI), folate (96 μg, 48% of RNI), magnesium (88 mg, 29% of RNI), potassium (950 mg, 27% of RNI), iron (5.3 mg, 36% of RNI), copper (0.49 mg, 41% of RNI), and selenium (40 μg, 53% of RNI). In contrast, the beef lasagne contained substantially higher amounts of riboflavin (0.5 mg, 35% of RNI), calcium (420 mg, 60% of RNI) and iodine (49 μg, 35% of RNI) than the other lasagne recipes. A breakdown of the nutritional composition of the different lasagne recipes is provided in Appendix C (Figure A2).

##### Chilli Recipes

The most nutrient-dense chilli recipe was the whole-food vegan chilli (NRF 17.3 score 480). The beef chilli was about 30% less nutrient dense (NRF 17.3 score 334), and the vegetarian chilli was 65% less nutrient dense (NRF 17.3 score 171) than the whole-food vegan version. A breakdown of the nutritional composition of chilli recipes is shown in Appendix C (Figure A3). The beef chilli had the highest protein content (28 g, 56% of RNI), followed by the vegetarian chilli (24 g, 48% of RNI) and the plant-based chilli (12 g, 24% of RNI). Both the whole-food vegan and vegetarian chillies provided over 30% of the daily recommended fibre intake (approximately 16 g per serving), while the beef chilli provided only 5 g (17% of RNI). The whole-food vegan chilli had the lowest saturated fat content per portion (0.4 g, 2% of RDI), compared to the vegetarian chilli (1.5 g, 8% of RDI) and the beef chilli, which exceeded the 30% RDI threshold with 7 g (35% of RDI). The whole-food vegan chilli also provided nearly double the RNI of vitamin A (retinol eq.) (1347 μg, 192% of RNI). In contrast, the vegetarian and beef chilli provided only 9% and 7% of the RNI for Vitamin A (retinol eq), respectively. Only the whole-food vegan chilli exceeded 30% of the RNI for vitamin C (70% of RNI), thiamine (B1) (49% of RNI) and potassium (31% of RNI). None of the chilli recipes met 30% of the RNI for folate, calcium, magnesium, and copper, although the whole-food vegan chilli provided considerably more of these micronutrients than the vegetarian or meat-based recipe versions. In contrast, only the beef chilli provided at least 30% of the RNI of vitamin B12 (1.4 μg, 93% of RNI) and zinc (5.1 mg, 54% of RNI). Lastly, all chilli recipes were low in iodine and riboflavin (B2).

##### Teriyaki Recipes

The whole-food vegan teriyaki was the most nutrient-dense teriyaki recipe, with an NRF 17.3 score of 467. The other teriyaki recipes had substantially lower nutrient densities (NRF 17.3 score 207 for vegan teriyaki and 159 for chicken teriyaki). A breakdown of the nutritional composition of teriyaki recipes is provided in Appendix C (Figure A4). The chicken teriyaki contained 33 g of protein (66% RNI), the whole-food vegan teriyaki provided 27 g (54% RNI), while the vegan teriyaki provided 13 g (26% RNI). Only the whole-food vegan teriyaki provided at least 30% of the RNI of fibre (12 g, 40% of RNI). The vegan and chicken teriyaki provided only 7% of the RNI for fibre. All teriyaki recipes were low in saturated fat, with values well below 30% of the RNI. Regarding micronutrients, only the whole-food vegan recipe exceeded 30% of RNI for vitamin A (retinol eq) (53% of RNI), vitamin C (75% of RNI), thiamine (B1) (87% of RNI), magnesium (35% of RNI) and copper (44% of RNI). The vegan teriyaki was the only recipe that provided sufficient amounts of calcium (50% of RNI) and iron (39% of RNI). None of the teriyaki recipes reached 30% of RNI for riboflavin, folate and potassium, though the whole-food vegan teriyaki provided more riboflavin, folate and potassium than the vegan or chicken teriyaki recipes. All teriyaki recipes were low in iodine.

##### Curry Recipes

The whole-food vegan curry was the most nutrient-dense curry recipe (NRF 17.3 score 394), followed by the vegan curry (NRF 17.3 score 309). The chicken curry was not only the least nutrient-dense curry recipe (NRF 17.3 score of 143) but also the least nutrient-dense among all the recipes examined in this study. This was due to the high amounts of nutrients to limit but relatively low amounts of nutrients to encourage. Except for protein (35 g, 70% of RNI), the chicken curry provided only 1–22% of the RNI for the rest of the micronutrients. In contrast, the plant-based curry recipes had considerably higher nutrient density. The whole-food vegan curry was particularly high in vitamin A (retinol eq) (781 μg; 112% of RNI). Both the vegan and the whole-food vegan recipes were high in folate, providing 93 μg (47% of RNI) and 64 μg (32% of RNI) of folate, respectively, while the chicken curry provided 6% of the RNI (12 μg). The plant-based curries were also high in vitamin E (43% of RNI of vitamin E) and vitamin C (140% of RNI). While plant-based curry recipes had higher nutrient density than the chicken curry, none of the recipes provided 30% of RNI for riboflavin, vitamin B12, calcium, magnesium, potassium, iodine, copper, selenium and iron. A breakdown of the nutritional composition of curry recipes is shown in Appendix C (Figure A5).

### 3.2. Environmental Impact

**Figure 3:**
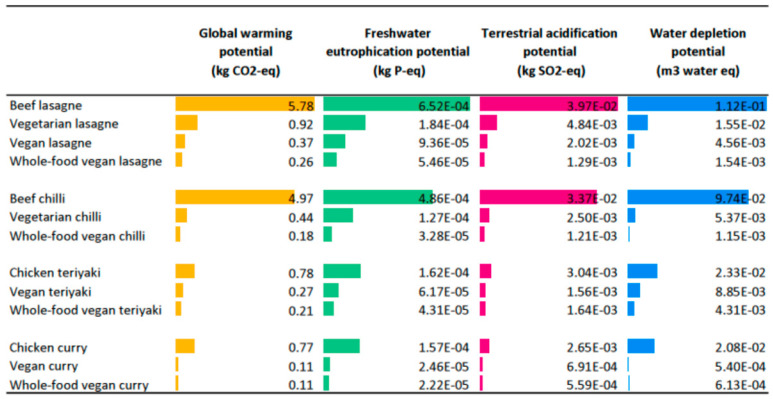
GWP, FEP, TAP, and WDP of lasagne, chilli, teriyaki, and curry meals and their meat-based, vegetarian, vegan, and whole-food vegan versions (adapted from Takacs et al., 2022 [15]).

The results of the life cycle assessment, detailed in the original study of Takacs et al. [15], showed that the vegan recipe variations of meals had substantially lower environmental impacts across all impact categories than their meat-based and vegetarian versions. Environmental impacts of meat-based meals were, on average, 14 times higher than those of vegan meals, while vegetarian meals had three times greater impact than vegan meals. Figure 3 shows a detailed breakdown of the results of the environmental impacts of recipes across the four impact categories.

**Figure 4:**
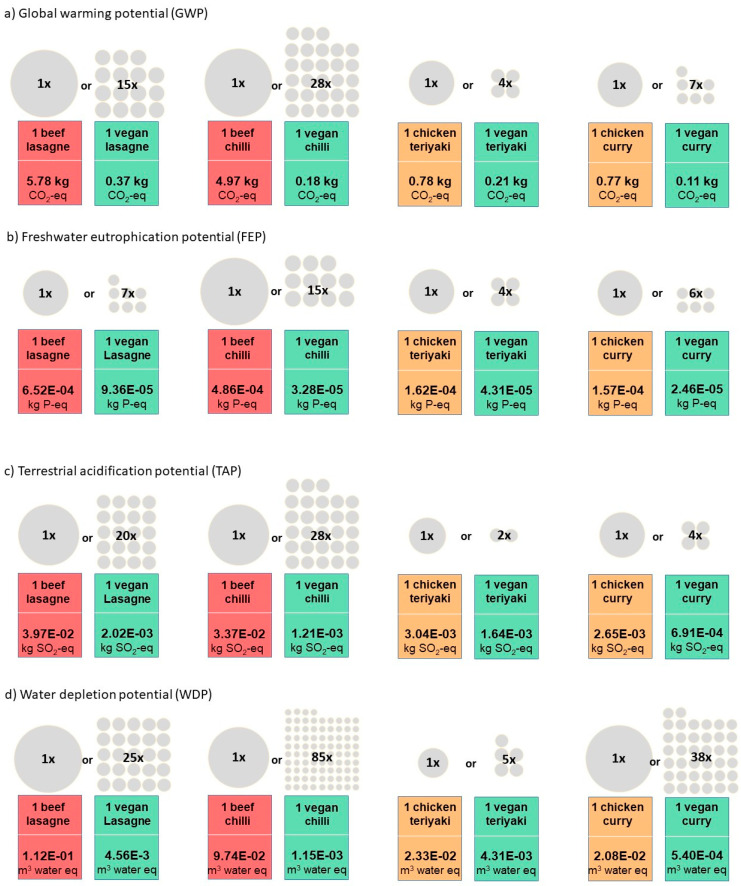
Comparison of the GWP, FEP, TAP and WDP of meat-based and plant-based versions of meals.

The magnitude of the differences in environmental impacts between animal- and plant-based recipes are illustrated in Figure 4 for each impact category. For example, the global warming potential (GWP) of one beef lasagne (5.78 kg CO_2_ eq) was equivalent to the GWP of 15 vegan lasagnes, while the GWP of one beef chilli (4.97 kg CO_2_ eq) was the same as 28 plant-based chillis. In other words, eating a plant-based chilli for lunch every day for an entire month would result in the same climate impact as eating one beef chilli. While chicken meals did not have as high a climate impact as beef meals, one chicken teriyaki (0.78 kg CO_2_ eq) had the same impact as four plant-based teriyakis, and one chicken curry (0.77 kg CO_2_ eq) had the same impact as seven vegan curries. Regarding other impact categories, plant-based recipe versions of meals had 4–15 times lower freshwater eutrophication potential, 2–28 times lower acidification potential, and 5–85 times lower water depletion potential compared to their meat-based counterparts.

### 3.3. Recipe Costs

**Figure 5:**
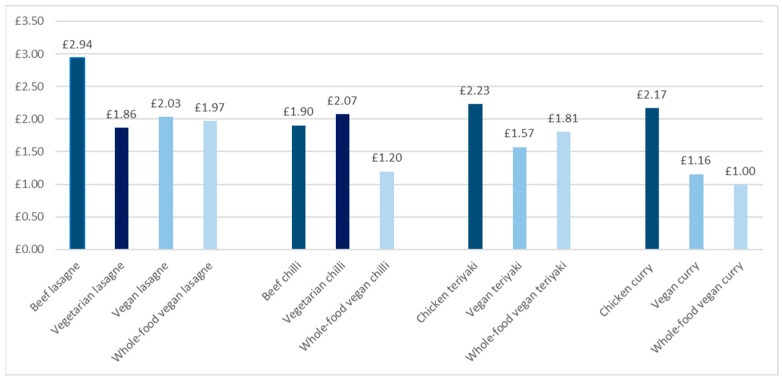
The prices of recipe variations of each meal. Prices are based on March 2025 prices.

Figure 5 shows the prices of recipe variations of each meal. In this study, the recipe cost of plant-based versions of meals was always less than that of their meat-based counterparts. The average recipe cost of meat-based recipes was the highest (GBP 2.31), followed by vegetarian recipes (GBP 1.97). In contrast, plant-based recipes (vegan and whole-food vegan) had the lowest average recipe cost (GBP 1.49).

In meat-based recipes, meat was the single biggest contributor to recipe costs, with chicken contributing between 63% and 75% and beef contributing between 46% and 70% of the total recipe cost. The primary cost contributors in vegetarian recipes were Quorn mince (25–63%) and cheese (16%), while in vegan recipes, it was the vegan meat substitutes (32–56%) and vegan cheese (11%) that contributed the most to the total recipe cost. In whole-food vegan recipes, the contribution of main ingredients (i.e., various vegetables, pulses, and pasta/noodles) to total recipe cost was relatively equally distributed.

### 3.4. Integrated Assessment

In the previous sections, the results of the nutritional quality, environmental impacts, and recipe costs of meals and their recipe variations were discussed individually. In this section, the results are integrated to identify the most sustainable meals (i.e., those with both low environmental impact and high nutrient density), while also exploring potential trade-offs between environmental impacts, nutritional quality, and recipe costs.

**Figure 6:**
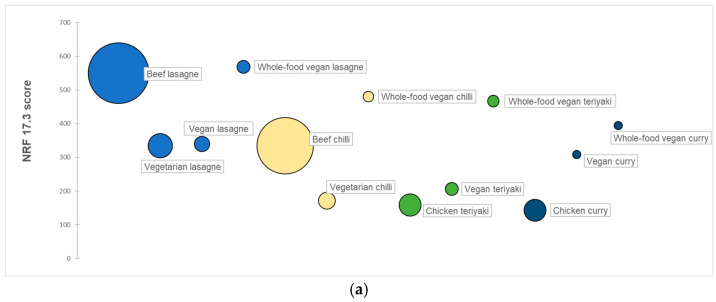

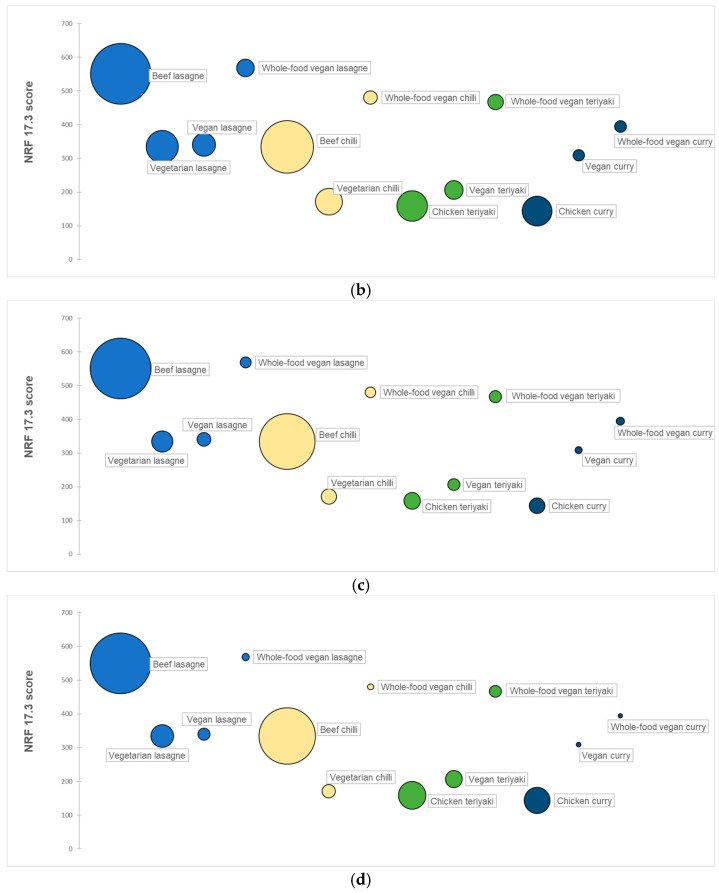
(**a**) NRF 17.3 scores of meals expressed in relation to their global warming potential (GWP). (**b**) NRF 17.3 scores of meals expressed in relation to their freshwater eutrophication potential (FEP). (**c**) NRF 17.3 scores of meals expressed in relation to their terrestrial acidification potential (TAP). (**d**) NRF 17.3 scores of meals expressed in relation to their freshwater depletion potential (WDP). The size of the circles (area) represents the magnitude of GWP, FEP, TAP or WDP of the meals respectively, while along the y-axis, the NRF 17.3 scores of meals are shown.

Figure 6 shows the nutrient density (NRF 17.3 scores) of meals relative to their (a) global warming, (b) freshwater eutrophication, (c) terrestrial acidification, and (d) water depletion potentials. The size of the circles corresponds to the magnitude of the environmental impact: the larger the circle, the higher the impacts. The *y*-axis shows nutrient density, with higher NRF 17.3 scores indicating more nutrient-dense meals. Lastly, the different colours correspond to the different dish types. Figure 6 shows that plant-based recipe variations of meals made with whole-foods had the lowest environmental impact (smallest circles) among the recipe variations of meals and the highest nutrient density (towards the top of the axis), regardless of the dish type or impact category.

**Figure 7:**
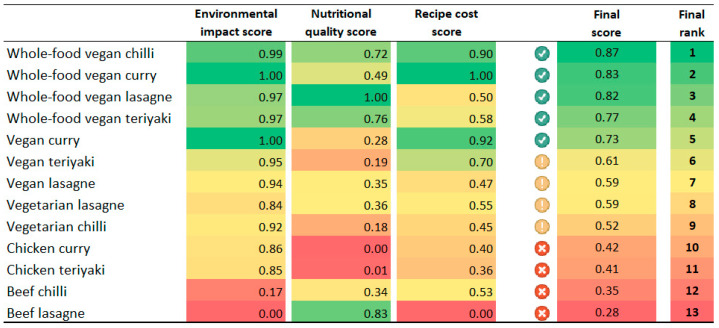
Ranking of meals relative to each other based on their final normalised scores obtained from their environmental impact, nutritional quality and recipe cost scores. Meals are ranked and colour coded relative to each other. In each criterion, 1.00 is given to the meal with the best performance (green), while 0.00 is given to the meal with the worst performance (red). Note: The final normalised scores are only shown to two significant figures; hence, the slight differences in values between certain meals are not visible due to the rounding.

Figure 7 shows the ranking of meals relative to each other based on their final normalised environmental impact score, nutritional quality score and recipe cost score when all criteria were weighted equally. Meals with the highest scores are shaded in green, while meals with the lowest scores are shaded in red in each criterion. The results show that plant-based recipe versions of the meals consistently achieved the highest overall sustainability scores (i.e., final scores), demonstrating their superior performance over their meat-based or vegetarian versions.

The results in Figure 7 also show trade-offs between the environmental, nutritional, and economic outcomes of a given meal. Whether a trade-off exists depends on the specific context of the comparison. For example, while the beef lasagne had the worst environmental score and was the most expensive recipe in this study (hence the normalised scores of 0.00), it was the second most nutritious meal after the whole-food vegan lasagne. However, when comparing only lasagne recipes, a trade-off is not present as the whole-food vegan lasagne offers both higher nutrient density and significantly lower environmental impact while also having a lower recipe cost than the beef version. However, when comparing beef lasagne to its vegetarian or standard vegan variations, a trade-off emerges in terms of nutritional quality. In terms of costs, the individual assessment revealed that the vegetarian lasagne was the cheapest lasagne recipe; however, when looking at the results of the integrated assessment, it becomes evident that with an additional GBP 0.11 per meal, a whole-food vegan version can be prepared that has 3.6 times lower GWP, 3.4 times lower FEP, 3.8 times lower TAP, 10.0 times lower WDP, and twice the nutrient density (NRF 17.3 score) of the vegetarian lasagne.

**Figure A1:**
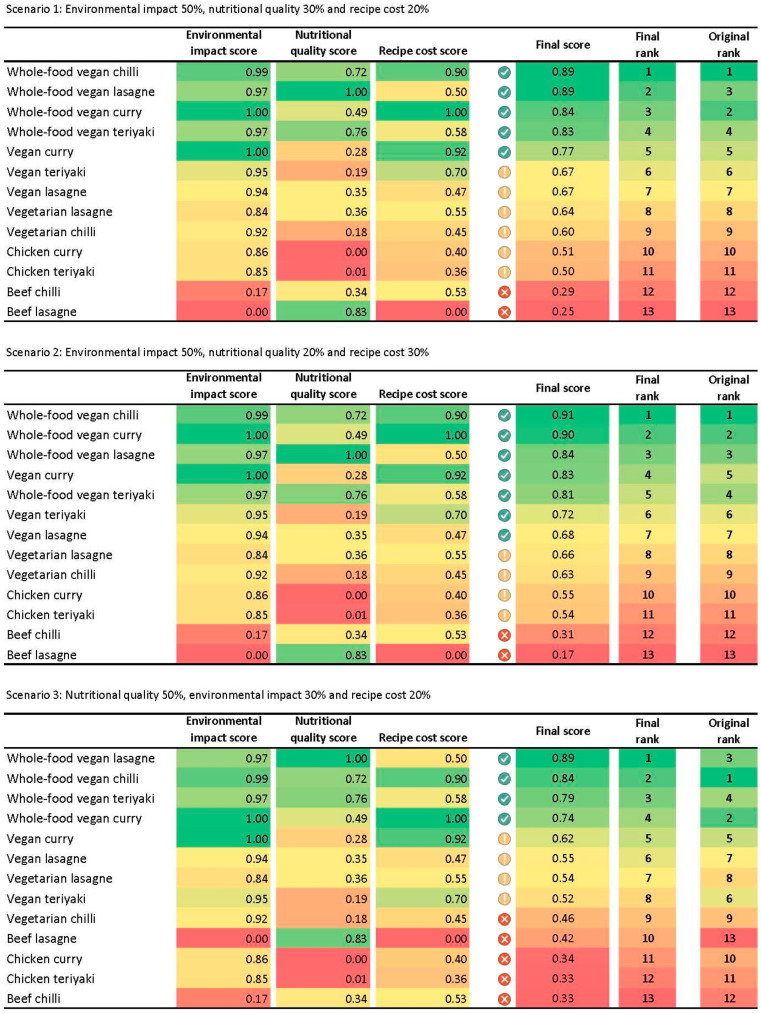

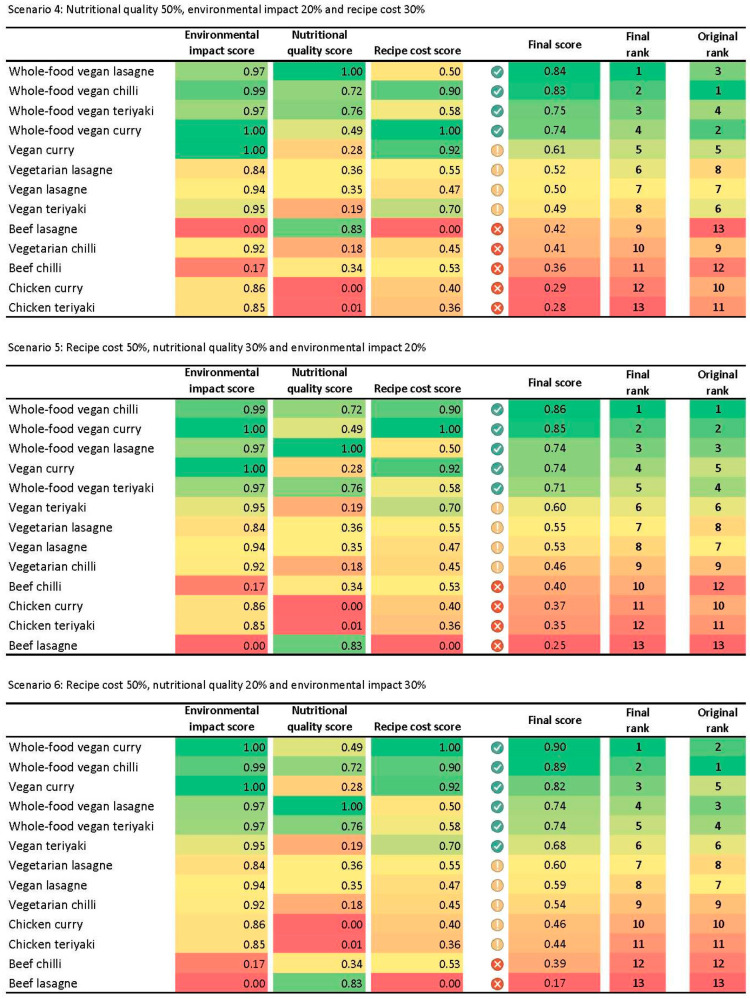
Ranking of meals relative to each other under the six different weighting scenarios. Note: ‘original rank’ refers to the rank given to meals when each criterion was weighted equally (baseline scenario). Meals are ranked and colour coded relative to each other, in each criterion, 1.00 is given to the meal with the best performance (green), while 0.00 is given to the meal with the worst performance (red). Note: The final normalised scores are only shown to two significant figures; hence, the slight differences in values between certain meals are not visible due to the rounding.

**Figure A2:**
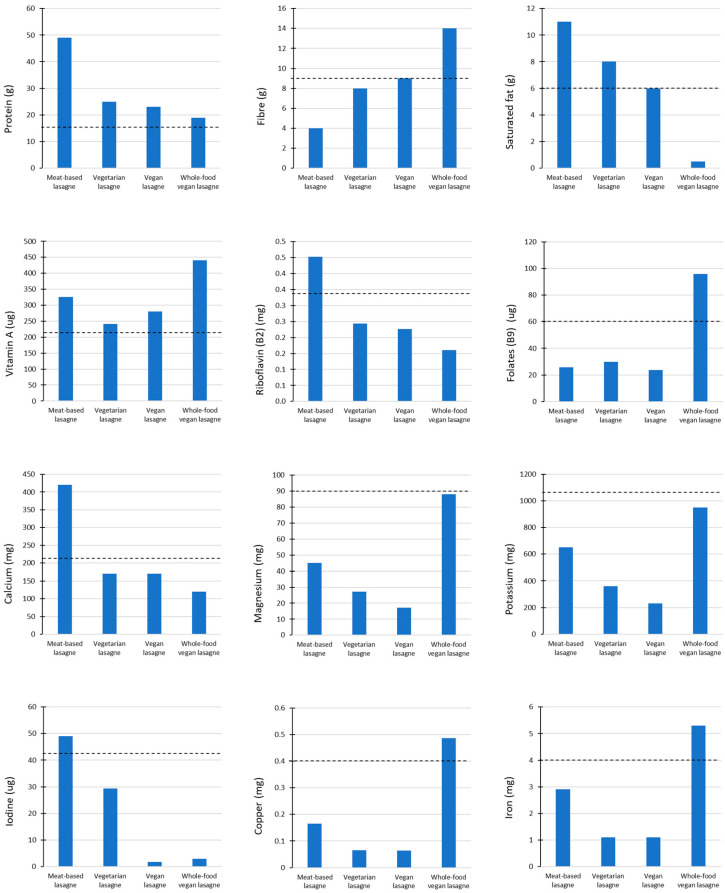
Detailed breakdown of the nutritional composition of meat-based, vegetarian, vegan and whole-food vegan lasagne recipes. Dashed line shows 30% of RNI of a given nutrient.

**Figure A3:**
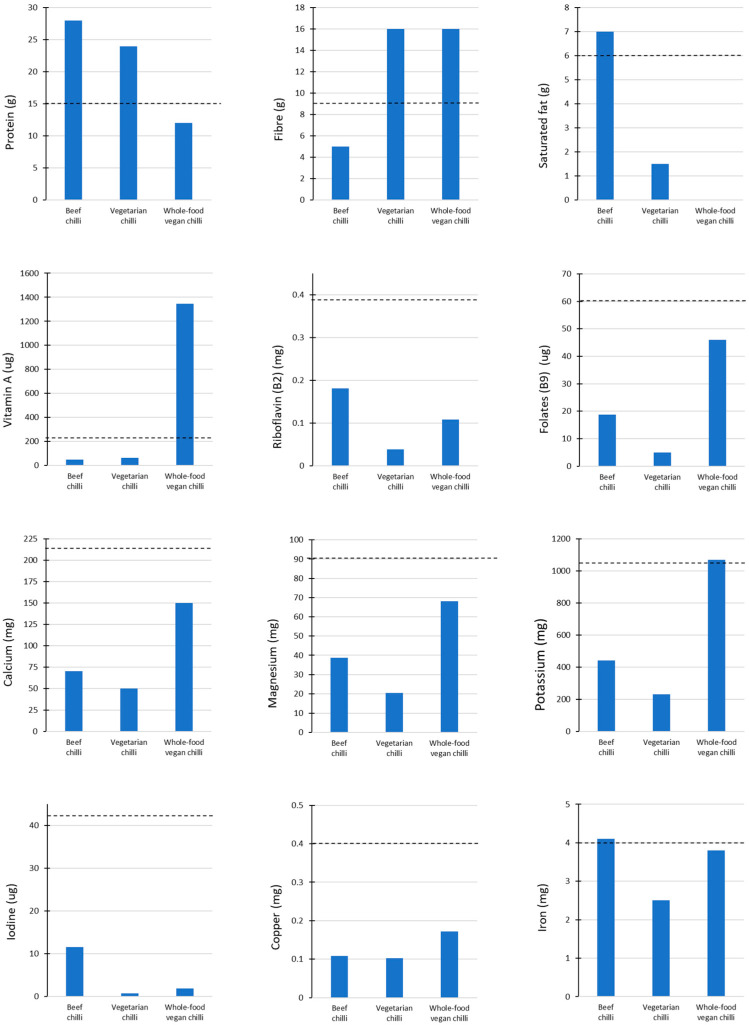
Detailed breakdown of the nutritional composition of meat-based, vegetarian, vegan and whole-food vegan chilli recipes. Dashed line shows 30% of RNI.

**Figure A4:**
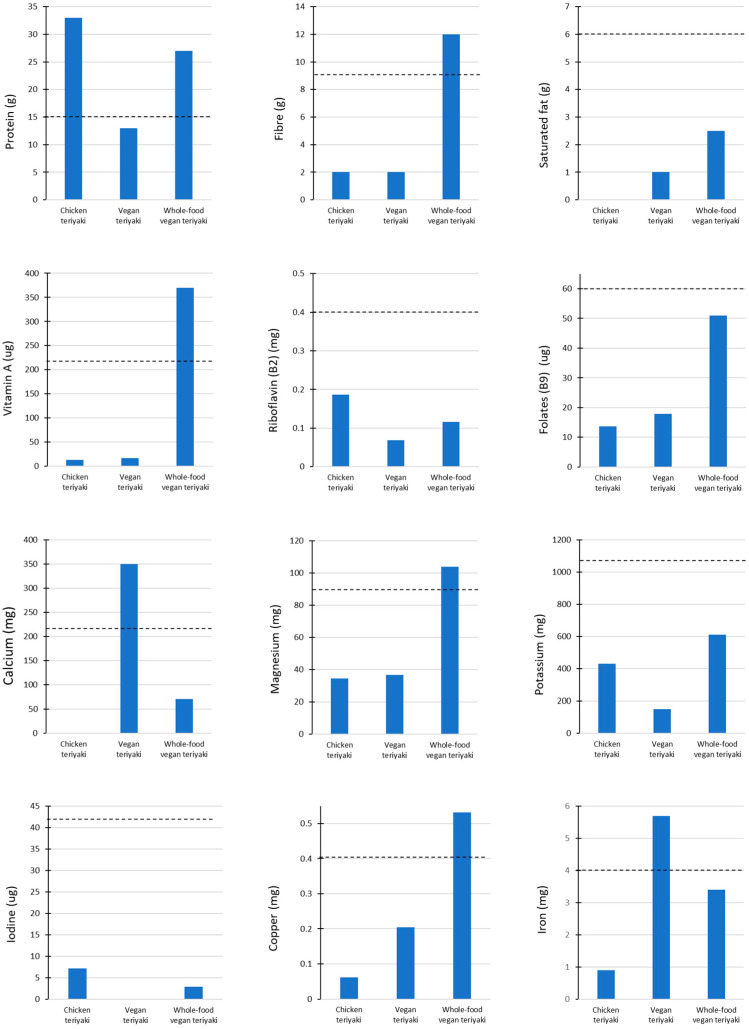
Detailed breakdown of the nutritional composition of meat-based, vegetarian, vegan, and whole-food vegan teriyaki recipes. Dashed line shows 30% of RNI.

**Figure A5:**
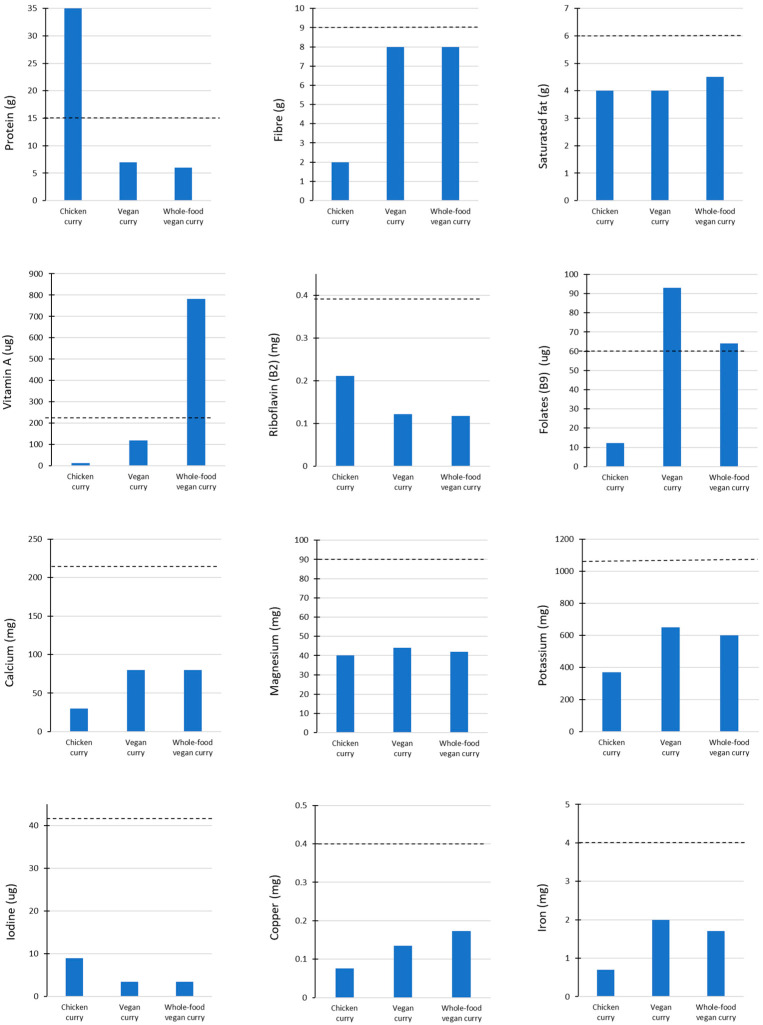
Detailed breakdown of the nutritional composition of meat-based, vegetarian, vegan, and whole-food vegan curry recipes. Dashed line shows 30% of RNI.

The results presented in this section are based on the assumption that all three criteria (environmental impact, nutritional quality, and cost) were equally important in determining the overall sustainability of meals. Depending on the values and priorities of food service providers and consumers, one criterion may be considered more important than the others. Nevertheless, when applying different weightings for each criterion, the overall ranking of the meals did not change considerably (see Appendix B, Figure A1). The top most sustainable meals remained the same plant-based meals, although their order varied with different weightings. Similarly, the least sustainable meals were consistently those made with animal-based ingredients.

In summary, this integrated assessment found that plant-based recipe variations of meals were the most sustainable, offering lower costs, reduced environmental impact, and higher nutritional quality. Integrated assessments are not only useful for complementing individual assessments, but they are also vital for identifying potential trade-offs between cost, nutrition, and environmental impact, and for enabling informed, evidence-based decision-making. Given the interconnections between environmental, social, and economic aspects of food choices, a comprehensive approach is crucial. The relevance and implications of these findings are discussed in the next section.

## 4. Discussion

### 4.1. Towards Healthy and Sustainable Meals

This study highlighted the potential of ingredient substitutions at the meal level to not only reduce the environmental impact but also to maintain or improve the nutritional quality of meals while often reducing costs. As demonstrated above, meat-based meals in this study with high environmental impact were transformed into more sustainable alternatives by replacing animal-based ingredients (e.g., meat and dairy) with plant-based options. In this study, plant-based ingredients significantly reduced the environmental impact of meals in all cases while also improving the nutrient density of recipes in most cases.

In the UK and other countries, a wide variety of plant-based meat and dairy alternatives are now available, providing a straightforward way to substitute animal-based ingredients in recipes without significantly altering the taste, texture or familiarity of meals. Our findings align with previous research indicating that plant-based substitutes can serve as healthful replacements for meat when chosen carefully [48]. Other studies also found that plant-based alternatives tend to be more environmentally sustainable and offer a favourable nutritional profile compared to their animal-based counterparts [49,50]. However, their nutritional quality is dependent on the type of product, formulation and degree of processing.

While substituting animal-based ingredients with their plant-based analogues may be a simple and straightforward strategy, the greatest improvements in nutritional quality in this study came from using whole and minimally processed plant-based ingredients in recipes (i.e., vegetables, legumes, whole grains, etc.). These findings are in alignment with previous research emphasising that healthy plant-based diets based on whole and minimally processed plant-based foods offer superior health benefits compared to those that rely on unhealthy or highly processed plant-based foods [36,51,52].

These findings highlight the potential benefits of increasing the availability and uptake of healthy plant-based meals in food service settings (e.g., restaurants, takeaways, cafeterias, schools, and workplaces) to reduce the environmental impact of food consumption while improving micronutrient intakes and public health. The food service sector can play a central role in shaping consumer choices and encouraging more sustainable dietary choices. The more convenient and accessible these healthy and sustainable options are, the more likely consumers are to choose them [53].

In addition to ingredient substitutions in recipes and offering healthy plant-based options, clear and accessible communication strategies are also crucial for guiding consumers toward sustainable food choices. Simple tools like traffic-light labels can effectively convey environmental and nutritional information, though practical implementation in food service settings remains a challenge [54,55,56].

Innovative menu and meal design has also been shown to drive plant-based meal uptake in food service settings. For example, making plant-based meals the “dish of the day” has resulted in 85% of participants choosing that dish over a menu with free choice [57]. Various nudge strategies can guide consumers toward nutritious, appetising, and sustainable meal options [53,58]. Saturating the choice environment with desirable plant-based options can also increase uptake, especially among meat-eaters [53].

Lastly, optimised descriptions that emphasise sensory appeal (e.g., taste, texture, and satisfaction) are simple yet cost-effective ways to increase plant-based meal consumption. Research shows that sensory-focused descriptions can enhance the appeal of plant-based options and boost their uptake in food service settings [59,60]. Describing meals in ways that highlight rewarding eating experiences can therefore be a crucial strategy for encouraging the shift towards the consumption of healthy and sustainable meals.

### 4.2. Strengths and Limitations

Integrated assessments are powerful tools to aid decision-making, particularly when synthesising results from different single-discipline approaches like life cycle assessment, nutrient profiling, and recipe cost. In this study, the integrated assessment enabled a comprehensive evaluation of how ingredient substitutions in different recipes influence the environmental impact, nutritional quality and cost of meals.

A key strength of this study is that it integrates three comprehensive assessments—nutritional quality, environmental impact, and cost—into a single analysis. By combining life cycle assessment, the Nutrient Rich Food Index, and recipe costing, this study moves beyond single-factor analyses and provides a more comprehensive evidence base for decision-making. Another strength lies in the use of real-life recipes from a university food service setting. As such, findings are grounded in real-world practices rather than hypothetical scenarios. The meals analysed—lasagne, chilli, teriyaki, and curry—were not only popular in the case study setting but are also widely consumed globally, increasing the broader relevance and applicability of the findings. While there may be regional variations in the ingredients and quantities used in recipes, the fundamental composition and structure of meals like lasagne, chilli, teriyaki, and curry remain broadly consistent across countries; otherwise, they would not be considered the same dishes.

Despite these strengths, the study has some limitations. While this analysis provided valuable insights into how ingredient substitutions can enhance meal sustainability, it included a limited number of recipes from a single food service provider. Furthermore, not every meat-based meal had a corresponding vegetarian or vegan alternative, as the study relied on existing recipes. Despite this, the findings remain relevant to similar institutional food service settings. In the UK alone, there are 405 higher education institutions with comparable food service operations, potentially serving approximately 2.9 million students [61], making the results applicable to a broader context. Future research could expand the sample size and include a more diverse range of meals from different cuisines to validate the trends.

Another limitation is around the normalisation method used in this study. While the min–max technique is effective for scaling results, this method cannot handle outliers well, as it only changes the range of the data, rather than reflecting the true magnitude of differences. For example, the beef recipes had significantly higher global warming potentials (5.78 kg CO_2_-eq for beef lasagne and 4.97 kg CO_2_-eq for beef chilli) compared to the other meals (all under 1 kg CO_2_-eq). Consequently, the values of the beef recipes were transformed into 0 and 0.14, while the values for the rest of the recipes were transformed into a narrow range between 0.86 and 1. As a result, the normalised values did not show clearly the real magnitude of differences between the environmental impacts of vegetarian and vegan recipes, even though the results of the life cycle assessment (Figure 3) demonstrated that vegetarian meals had, on average, three times higher environmental impacts than vegan meals. Nevertheless, this highlights how individual and integrated assessments can complement each other and be used side by side to provide a more nuanced understanding of the different sustainability aspects of meals and their recipe variations.

Another limitation is that the nutrient profiling model used in this study only assesses the nutrient density and nutrient composition of meals, disregarding aspects like nutrient bioavailability. The bioavailability of certain nutrients in plant-based foods, such as iron, zinc, and calcium, can be lower compared to animal-based foods due to differences in nutrient forms (e.g., heme vs. non-heme iron) and the presence of antinutrients (e.g., phytates, oxalates, and polyphenols). However, there are proven methods to increase the bioavailability of nutrients in plant-based foods. For example, combining various plant foods can improve iron absorption, with vitamin C being a well-known enhancer of non-heme iron absorption [62]. Notably, the plant-based recipes analysed in this study contained substantially higher levels of vitamin C than their animal-based counterparts, which likely supports improved non-heme iron absorption. However, such complexities are not captured by nutrient profiling. Differences in nutrient uptake between plant-derived and animal-based nutrients may therefore reduce the accuracy of the nutritional quality assessment of meals. Nutrition metrics in future research could benefit from the inclusion of measures of bioavailable/digestible nutrient fraction in addition to nutrient density [63].

Lastly, in this study equal weighting was assumed for environmental impact, nutritional quality, and cost in determining the overall sustainability of meals. However, in practice, different stakeholders may prioritise these factors differently. To address this, multiple weighting scenarios were developed and tested. While the order of rankings differed slightly under each scenario, plant-based meals consistently ranked as the most sustainable options, while animal-based meals as the least sustainable. Future assessments could also incorporate additional dimensions of food consumption, such as social and ethical considerations, to make the integrated assessment more comprehensive.

## 5. Conclusions

This study evaluated the nutritional quality, environmental impact and cost of four popular meals and their meat-based, vegetarian, and vegan recipe variations using an integrated assessment approach. The results indicated that replacing animal-based ingredients in recipes with whole and/or minimally processed plant-based alternatives can lower environmental impact while often enhancing nutrient density and reducing recipe costs. Although the sample size was limited, the real-world recipes analysed in this study are widely consumed, making the findings relevant to a wide range of audiences. However, the methodological limitations and sample size should be kept in mind when interpreting results and their generalisability. Future research may explore consumer acceptance of whole-food plant-based meals and the effectiveness of various interventions to improve the availability, accessibility, acceptability and uptake of healthy and environmentally sustainable meals in various settings. The food service sector plays a crucial role in promoting sustainable eating habits, and thus, studies are needed to assess the effectiveness of interventions that could help shift dietary patterns toward healthier and more sustainable choices.

## Data Availability

The data and results supporting the conclusions of this study are included in the article. Further enquiries can be directed at the corresponding author.

## Appendix A

**Table A1:** Meals analysed in this study with their ingredients, serving weights, and energy content per meal.

Meal	Ingredients	Weight of Recipe (g) Before Cooking	Weight of Recipe (g) After Cooking	Energy Content (kcal)
Beef lasagne	Beef mince, semi skimmed milk, chopped tomatoes, lasagne pasta sheets, mozzarella, cheddar cheese, onions, flour, margarine, celery, carrots, Italian style hard cheese, bacon, tomato paste, rapeseed oil, garlic, basil, cooking salt, black pepper, bay leaves.	504	589	650
Vegetarian lasagne	Chopped tomatoes, semi skimmed milk, lasagne pasta sheets, Quorn mince, cheddar cheese, onions, spinach, red peppers, green peppers, flour, margarine, garlic, rapeseed oil, tomato paste, cooking salt, bay leaf, black pepper.	409	432	490
Vegan lasagne	Chopped tomatoes, soya milk, vegan soya mince, lasagne pasta sheets, spinach, vegan cheese, red peppers, green peppers, margarine, flour, garlic, rapeseed oil, tomato paste, oregano, cooking salt, bay leaf, black pepper.	411	436	480
Whole-food vegan lasagne	Chopped tomatoes, cauliflower, lentils, aubergines, courgettes, lasagne pasta sheets, onions, spinach, red peppers, green peppers, carrots, unsweetened soya milk, garlic, sea salt, tomato paste, oregano, black pepper.	538	570	330
Beef chilli	Beef mince, chopped tomatoes, onions, kidney beans, tomato paste, garlic, cumin seeds, rapeseed oil, green chillies, chilli powder, beef stock cubes, red pepper hot sauce, black pepper, oregano, bay leaf.	402	331	310
Vegetarian chilli	Quorn mince, chopped tomatoes, kidney beans, onions, tomato paste, extra virgin olive oil, garlic, cumin seeds, chilli powder, green chillies, vegetable stock cube, red pepper hot sauce, black pepper.	359	266	230
Whole-food vegan chilli	Chopped tomatoes, kidney beans, sweet potatoes, carrots, courgettes, celery, onions, tomato paste, salt, garlic, chilli powder, green chilli peppers, cumin seeds, black pepper.	462	342	220
Chicken teriyaki	Diced chicken breast, noodles, teriyaki sauce, coriander, ginger puree.	253	215	280
Vegan teriyaki	Tofu, noodles, teriyaki sauce, rapeseed oil, corn flour, coriander, ginger puree.	243	219	310
Whole-food vegan teriyaki	Wholemeal noodles, tempeh, sweet potatoes, white cabbage, carrot, green cabbage, broccoli, red pepper, onion, tomatoes, teriyaki sauce, coriander, ginger.	333	300	510
Chicken curry	Chicken breast, Thai green concentrated sauce, rapeseed oil, onions, coriander, coconut milk powder.	273	232	240
Vegan curry	Broccoli, onions, aubergines, courgettes, Thai green concentrated sauce, beansprouts, rapeseed oil, garlic.	366	329	190
Whole-food vegan curry	Broccoli, onions, aubergines, courgettes, carrots, coconut milk, beansprouts, Thai green curry paste, garlic.	296	266	140

## Appendix B

Different weightings applied to test if the final ranking of meals and recipe variations would change. Results of the ranking of meals under the six different weighting scenarios are presented in Figure A1.

Baseline scenario: If all impacts are of equal priority (presented in the paper)
Environmental impact 33.3%, nutritional quality 33.3% and recipe cost 33.3%

Additional six different weighting scenarios
If the main priority is environmental impact:
Scenario 1: Environmental impact 50%, nutritional quality 30% and recipe cost 20%
Scenario 2: Environmental impact 50%, nutritional quality 20% and recipe cost 30%
If the main priority is nutritional quality:
Scenario 3: Nutritional quality 50%, environmental impact 30% and recipe cost 20%
Scenario 4: Nutritional quality 50%, environmental impact 20% and recipe cost 30%
If the main priority is recipe cost:
Scenario 5: Recipe cost 50%, nutritional quality 30% and environmental impact 20%
Scenario 6: Recipe cost 50%, nutritional quality 20% and environmental impact 30%

## Appendix C

**Table A2:** The NRF 9.3 and NRF 17.3 scores calculated per served meal. The maximum scores are 600 for NRF 9.3 and 1400 for NRF 17.3. Recipes that had an NRF score of at least 30% of the maximum score (>180 for NRF 9.3 and >420 for NRF 17.3) are highlighted in light green.

	NRF 9.3 per Meal	NRF 17.3 per Meal
**Lasagne recipes**		
Meat-based	251	550
Vegetarian	202	335
Vegan	236	340
Whole-food vegan	340	569
**Chilli recipes**		
Meat-based	114	334
Vegetarian	143	171
Whole-food vegan	360	480
**Teriyaki recipes**		
Meat-based	80	159
Vegan	140	207
Whole-food vegan	251	467
**Curry recipes**		
Meat-based	60	143
Vegan	202	309
Whole-food vegan	300	394

**Table A3:** Nutrient composition of meals. n/a—not applicable.

		Lasagne				Chilli			Teriyaki			Curry			RNI
		Beef	Vegetarian	Vegan	Whole-food vegan	Beef	Vegetarian	Whole-food vegan	Chicken	Vegan	Whole-food vegan	Chicken	Vegan	Whole-food vegan	
Serving size	g	589	432	436	570	331	266	342	215	219	300	232	329	266	n/a
Energy	kcal	650	490	480	330	310	230	220	280	310	510	240	190	140	2000
Carbohydrate	g	63	63	69	68	18	33	55	36	42	92	14	27	22	n/a
Fat	g	24	19	17	4	16	7	2	1	11	9	6	9	7	n/a
Protein	g	49	25	23	19	28	24	12	33	13	27	35	7	6	50
Fibre	g	4	8	9	14	5	16	16	2	2	12	2	8	8	30
Monounsaturated Fat	g	4.7	3.9	2.8	0.2196	6.9	2.7	0.2491	0.4108	4.6	1.1	0.6057	2.1	0.4842	36
Free sugars	g	1	0	0	0	1	1	0	0	0	0	0	0	0	30
Saturated fat	g	11	8	6	0.5	7	1.5	0	0	1	2.5	4	4	4.5	20
Sodium	mg	550	430	500	580	270	290	160	480	430	1020	620	590	200	1600
Potassium	mg	650	360	230	950	440	230	1070	430	150	610	370	650	600	3500
Calcium	mg	420	170	170	120	70	50	150	0	350	70	30	80	80	700
Magnesium	mg	45	27.2	17.1	88	38.6	20.5	68	34.5	36.7	104	40	44	42	300
Iron	mg	2.9	1.1	1.1	5.3	4.1	2.5	3.8	0.9	5.7	3.4	0.7	2	1.7	14.8
Vitamin A (Total RE)	μg	326	241	280	441	49	65	700	13.5	17	370	13.7	120	700	700
Vitamin E	mg	2.2	0.9244	2.3	0.8539	0.7163	0.5004	0.8941	0.167	1.1	0.8194	0.2138	1.7	1.7	4
Vitamin C	mg	6.9	33.4	32.1	40	3.4	4.4	28	0.54	1.8	30.1	0.9169	40	40	40
Vitamin D	μg	1.4	0	1.4	0	0.6	0	0	0	0	0	0	0	0	10
Thiamin (B1)	mg	0.242	0.1075	0.0852	0.4549	0.188	0.1057	0.486	0.1829	0.136	0.8666	0.1532	0.2513	0.2436	1
Riboflavin (B2)	mg	0.4524	0.2436	0.227	0.1599	0.1817	0.0383	0.108	0.186	0.0682	0.1162	0.2116	0.1221	0.1179	1.3
Folates (B9) Total	μg	25.9	29.9	23.7	96	18.8	4.9	46	13.6	17.9	51	12.2	93	64	200
Vitamin B12 (Cobalamin)	μg	1.5	0.7866	0.8246	0.0288	1.4	0	0	0.0008	0	0	0.0007	0	0	1.5
Iodine (I)	μg	49	29.3	1.7	2.9	11.6	0.6901	1.9	7.2	0.0001	2.9	9	3.4	3.4	140
Copper (Cu)	mg	0.1652	0.0651	0.0644	0.4861	0.1085	0.1022	0.1714	0.0614	0.204	0.5321	0.0762	0.1348	0.1727	1.2
Zinc (Zn)	mg	6.1	0.7021	0.3341	2.4	5.1	0.3195	1.3	0.946	0.9261	2.8	0.9558	0.937	0.902	9.5
Selenium (Se)	μg	11.1	1.6	0.6318	40	10.4	0.2813	8.4	14.4	8.9	14.1	16.8	2.1	1.8	75
